# Unlocking Biomedical Potential of the [Cu(l‑isoleucine)(1,10-phenanthroline)(H_2_O)]Cl·2H_2_O Complex: A Comprehensive Study of Synthesis, Spectroscopic Analyses, Antitumor Efficacy, and Computational Insights

**DOI:** 10.1021/acsomega.6c00071

**Published:** 2026-05-14

**Authors:** Marinaldo V. de Souza Junior, Jayson C. dos Santos, Jad L. F. Simplicio, João G. de Oliveira Neto, Camila P. S. Silva, Aramys S. Reis, Adenilson O. dos Santos, Alejandro P. Ayala, Eliana B. Souto, Francisco F. de Sousa

**Affiliations:** † Center for Sciences of Imperatriz, Federal University of Maranhão − UFMA, 65900-410 Imperatriz, MA, Brazil; ‡ UCD School of Chemical and Bioprocess Engineering, 8797 University College Dublin, Belfield, Dublin 4 D04 V1W8, Ireland; § Laboratory of Pathophysiology and Therapeutic Research, Center for Social Sciences, Health and Technology, Federal University of Maranhão – UFMA, 65900-410 Imperatriz, MA, Brazil; ∥ Department of Physics, Federal University of Ceará − UFC, 65455-900 Fortaleza, Ceará, Brazil; ⊥ Institute of Exact and Natural Sciences, Federal University of Pará − UFPA, 66075-110 Belém, PA, Brazil

## Abstract

We report the synthesis and comprehensive characterization of a ternary copper­(II) complex, [Cu­(l-isoleucine)­(1,10-phenanthroline)­(H_2_O)]­Cl·2H_2_O, combining single-crystal X-ray diffraction (SCXRD), ultraviolet–visible (UV–vis), Fourier-transform infrared (FT-IR), and Raman spectroscopy, and Hirshfeld surface analyses, with molecular docking, and *in vitro* cytotoxicity assays. SCXRD reveals a distorted square-pyramidal Cu­(II) center (monoclinic, *P*2_1_ (*C*_2_^2^) space group, 300 K). Hirshfeld analysis indicates that H···H (52.8%), O···H/H···O (17.4%), and Cl···H/H···Cl (10.3%) contacts dominate the crystal packing. Docking of the biologically main cation [Cu­(l-Ile)­(phen)­(H_2_O)]^+^ predicts favorable binding to deoxyribonucleic acid (Δ*G* = −8.26 kcal/mol) and bovine serum albumin (Δ*G* = −7.17 kcal/mol), supported by π–π and electrostatic interactions. The complex exhibits a dose-dependent antiproliferative effect against SiHa cervical carcinoma cells (IC_50_ = 2.57 μM) and comparable toxicity toward GM07492A fibroblasts (IC_50_ = 2.77 μM), yielding a selectivity index of 1.08. These findings establish a structurally validated Cu­(II) mixed-ligand complex and provide an integrated experimental-computational framework for future optimization toward improved biological selectivity.

## Graphical abstract

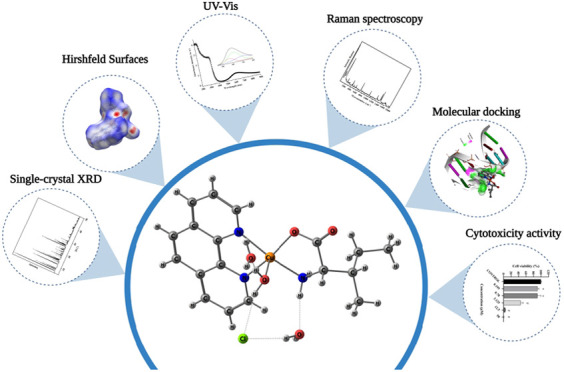

## 1 Introduction

The use of amino acids in the formation of ternary complexes with metal ions, such as copper­(II) (Cu­(II)) and *N,N*-donor aromatic ligands, is an effective strategy to generate new drug candidates by modulating the physicochemical and biological properties of these systems.^1–8^ In particular, amino acids, such as l-isoleucine (l-Ile), offer not only chirality but also promote increased solubility and stability of the complexes, favoring the interaction with essential biomolecules, such as deoxyribonucleic acid (DNA).^9,10^

Ternary complexes that combine amino acids and aromatic ligands, such as 1,10-phenanthroline (phen), have shown great potential in biological processes, especially in oxidative DNA cleavage and antibacterial activity.^2–6^ Incorporation of amino acids into complexes with Cu­(II) ions enables the adjustment of both the DNA-binding affinity and the capacity to generate reactive oxygen species (ROS), which are responsible for the oxidative cleavage of genetic material. This type of modification can significantly improve the performance of complexes in cleavage processes, making them viable therapeutic candidates for the development of new artificial nucleases.^11,12^

The presence of phen gives the complex a rigid and planar geometry, ideal for intercalation between DNA base pairs, while also facilitating electron-transfer mechanisms, which are essential for inducing controlled oxidative damage to genetic material.^13,14^ The synergistic combination of the high coordinating capacity of Cu­(II), the affinity of phen for hydrophobic regions of DNA, and the bioactive properties of l-Ile allows the development of complexes with superior performance compared to binary systems.^4^

The introduction of chiral amino acids into these complexes also opens up new perspectives regarding stereospecific selectivity in biomolecular interactions, which may result in more targeted and less damaging cytotoxic effects on healthy cells.^15,16^ Thus, ternary complexes containing Cu­(II), phen, and l-Ile represent a versatile and promising platform for the synthesis of a new generation of antitumor agents with multiple mechanisms of action, including DNA intercalation, oxidative cleavage, and apoptosis induction.

Chikira et al.^11^ studied the orientation of [Cu­(phen)]^2+^ complexes and their ternary complexes with amino acids, in their interaction with DNA, using electron paramagnetic resonance (EPR) spectroscopy. Results confirmed that these complexes bind to DNA through different modes, including intercalative and nonintercalative interactions. Intercalation was favored by strategically positioned methyl groups on the phen ring, but hindered by substituents that compromise the planarity of the molecule. Ternary complexes containing amino acids, such as glycine, leucine, serine, threonine, cysteine, methionine, and asparagine, exhibited a partial replacement of the amino acids by DNA-coordinating groups. In contrast, complexes with lysine, arginine, and glutamine remained structurally intact. Intercalative binding was more pronounced in the complexes that maintained greater planarity and presented specific interactions with the DNA.

Rodrigues et al.^4^ reported that Cu­(II) complexes with phen and the amino acids l-asparagine and l-methionine had significant cytotoxic activity against prostate cancer (DU-145 and PC-3), breast cancer (MDA-MB-231 and MCF-7), and melanoma (MV3) cell lines. The complexes containing phen were more effective than those composed only of copper and l-asparagine, highlighting the crucial role of phen in potentiating cellular chemosensitivity. Souza Junior et al.^3^ reported that the ternary [Cu­(Glutamine)­(phen)­(H_2_O)]­NO_3_·H_2_O complex showed significant cytotoxic effects against prostate (PC-3) and glioblastoma (SNB-19) cancer cells. This activity was evaluated through *in vitro* assays, indicating the potential of the complex as a promising antitumor agent. Furthermore, Ramos et al.^5^ described the cytotoxic effect of the [Cu­(Serine)­(phen)­(H_2_O)]­NO_3_ complex against the human colorectal carcinoma cell line (HCT-116), revealing a significant cytotoxic effect. The selectivity index obtained suggests that this complex has the potential to minimize the adverse effects on healthy cells.

Although a closely related nitrate analogue, [Cu­(phen)­(l-Ile)­(H_2_O)]­NO_3_, has been reported in the crystallographic literature,^17^ its study was limited to synthesis/structure and did not address quantification of supramolecular contacts, biomacromolecular interaction modeling, or anticancer performance under *in* *vitro* conditions. In contrast, the present chloride-containing solid form enables a distinct hydrogen-bonding landscape (notably O–H···Cl contacts mediated by lattice/axial water) that can be quantitatively assessed by Hirshfeld surface analysis and connected to molecular recognition hypotheses. Thus, the novelty of this work lies in (*i*) establishing the chloride-dependent crystal packing and intermolecular contact profile, and (*ii*) integrating structure-validated modeling (DNA/BSA docking using the relevant cationic species) with antiproliferative screening, thereby providing a coherent structure–interaction–activity framework to guide future metallodrug optimization.

Rather than expanding a library of closely related derivatives, the present study focuses on establishing a detailed structure–interaction–activity relationship for a single, well-defined Cu­(II) mixed-ligand system. By integrating crystallographic validation, supramolecular analysis, biomolecular docking using physiologically relevant cationic species, and *in vitro* cytotoxic screening, this work provides a level of mechanistic and structural correlation rarely achieved in single-compound studies.

In this context, this work describes the first time synthesis of a new ternary aqua-(l-isoleucine-*N*,*O*)-(1,10-phenanthroline)-copper­(II) chloride dihydrate complex, named [Cu­(l-Ile)­(phen)­(H_2_O)]­Cl·2H_2_O, and its structural and spectroscopic characterization. Hirshfeld surface analyses were also performed together with molecular docking and the assessment of cytotoxic activity in two distinct cell lines. The results obtained provide important insights for advancing the rational design of metallodrug complexes, contributing to the development of new therapeutic strategies based on bioactive coordination systems.

## 2 Experimental and Theoretical Methodology

### 2.1 Crystal Growth

Phen and l-Ile were purchased commercially and used without further purification; no ligand synthesis was performed.

The ternary Cu­(II) complex was synthesized in aqueous-methanolic solution using a stepwise ligand addition protocol. First, CuCl_2_·2H_2_O (170 mg, 1 mmol, Sigma-Aldrich, St. Louis, MO, ≥99% purity) was dissolved in 15 mL of deionized water (Permution, Curitiba, PR, Brazil) under magnetic stirring at room temperature to obtain a clear blue solution of Cu^2+^ ions. Subsequently, monohydrate phen (396 mg, 2 mmol, Synth, Labsynth, SP, Brazil, 99% purity) was dissolved separately in 10 mL of methanol (Synth, Labsynth, SP, Brazil, 98.9% purity) and added dropwise to the copper­(II) solution under continuous stirring. The use of two equivalents of phen relative to Cu­(II) ensures the complete saturation of the equatorial coordination sites at this stage, forming the *bis*-chelate intermediate [Cu­(phen)_2_]^2+^, in which both phen molecules coordinate through their pyridinic nitrogen atoms (*N*,*N*-donors) and promotes full displacement of the coordinated water molecules from the Cu­(II) equatorial plane.

In a second step, l-Ile (140 mg, 1 mmol, Sigma-Aldrich, St. Louis, MO, ≥98.5% purity) was dissolved in 5 mL of deionized water and added dropwise to the [Cu­(phen)_2_]^2+^ solution under stirring. The pH of the resulting mixture was then adjusted to approximately 7 by the dropwise addition of NaOH solution (0.1 M, Sigma-Aldrich, ≥98% purity), promoting the partial deprotonation of the carboxyl group of l-Ile while preserving the protonation of the amine group. Under these conditions, the zwitterionic l-Ile species bearing a free carboxylate (COO^–^) and an unprotonated amine nitrogen (NH_2_) acts as a competitive bidentate *N*,*O*-chelator and displaces one phen molecule from the [Cu­(phen)_2_]^2+^ intermediate. The five-membered metallocycle formed upon l-Ile coordination, together with entropic and solvation effects, drives the substitution equilibrium toward the ternary product. The remaining axial coordination site is occupied by a water molecule (*O*-donor). The displaced phen molecule remains in solution and is not incorporated into the crystalline phase. The final Cu:phen:l-Ile molar ratio in solution was 1:2:1, with a total reaction volume of 30 mL. The overall reaction can be formally described as

$${\mathrm{CuCl}}_{2}\cdot 2H_{2}O+2\cdot \mathrm{phen}+L‐\mathrm{Ile}\to \left[\mathrm{Cu}\left(L‐\mathrm{Ile}\right)\left(\mathrm{phen}\right)H_{2}O)\right]\mathrm{Cl}\cdot 2H_{2}O+\mathrm{phen}+{\mathrm{Cl}}^{-}$$

The resulting blue solution was filtered through a 0.45 μm membrane to remove any undissolved material and transferred to an open crystallization vessel. Slow solvent evaporation at 35 °C over approximately 7–10 days afforded blue single crystals of [Cu­(l-Ile)­(phen)­(H_2_O)]­Cl·2H_2_O suitable for SCXRD analysis. A simplified representation of this formation pathway is also provided in Scheme 1.

**Scheme 1:**
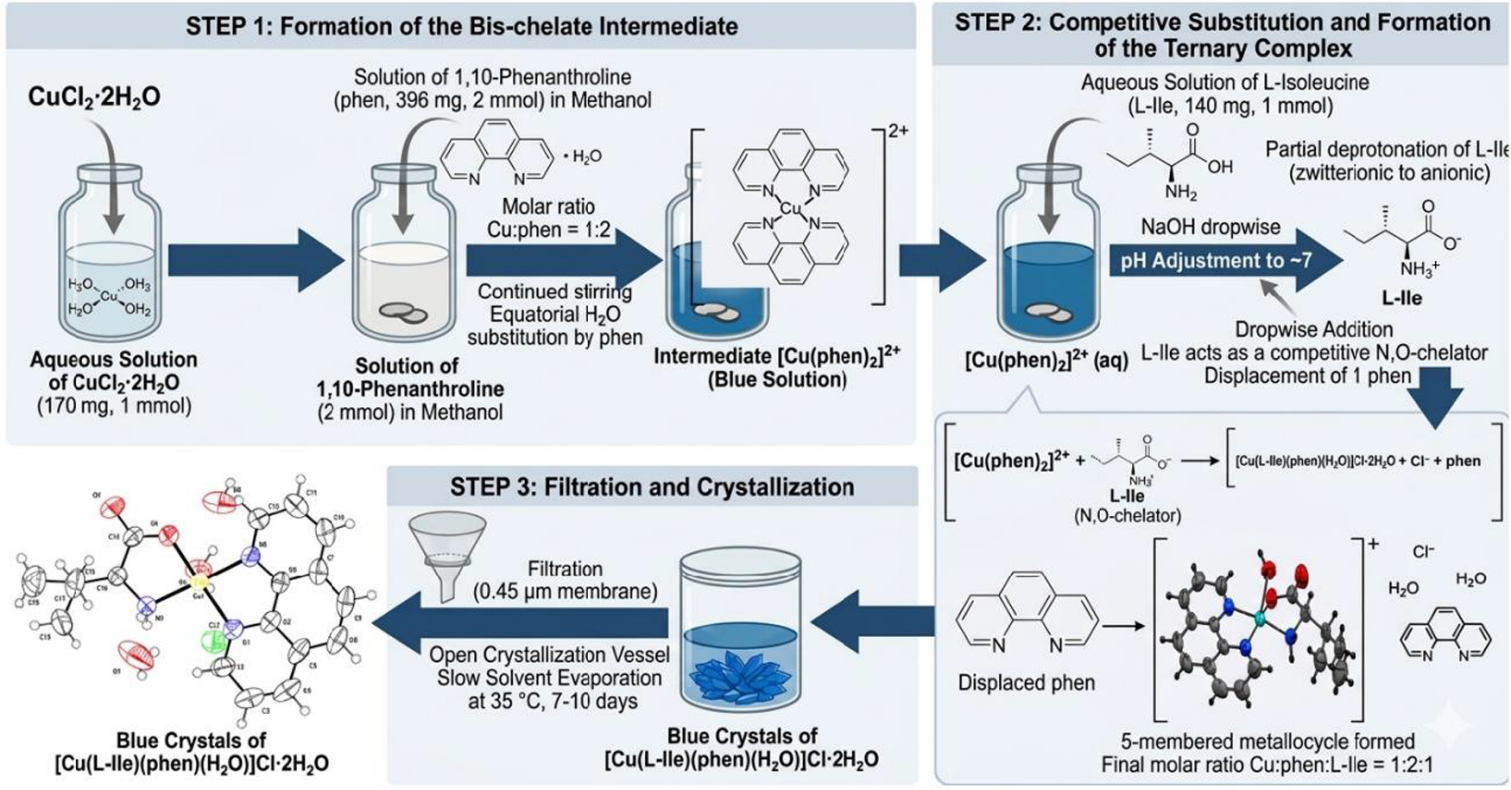
Synthetic Pathway and Crystallization Mechanism of the Complex [Cu­(l-Ile)­(phen)­(H_2_O)]­Cl·2H_2_O

### 2.2 SCXRD Methodology

SCXRD data were collected on a Bruker D8 Venture J-geometry diffractometer (Bruker, Berlin, Germany) equipped with a Photon II CPAD detector and an Incoatec IlS 3.0 Mo Kα microfocus source (λ = 0.71073 Å). APEX III software was used to determine the unit cell and collect the data. Data reduction and global cell refinement were performed using the Bruker SAINT software package,^18^ and a multiscan absorption correction was applied using the SADABS program.^19^ The structure was solved using ShelXT software, employing intrinsic/direct phasing methods, and refined using ShelXL, which utilized the full-matrix least-squares method in *F* ^2^ via the Olex2 graphical interface.^20^ The hydrogen atoms were not anisotropically refined, as they were positioned according to geometric criteria and treated using the riding model.^19^ The MERCURY^21^ and ORTEP 3 ^22^ programs were used to prepare the crystallographic information file (CIF) and generate the graphical representations for publication. The CIF file has been deposited into the Cambridge Structural Database (CSD) under code 2372426 (free copies of the data are available at the following link: https://www.ccdc.cam.ac.uk/structures/). Other structural parameters are presented in Tables S1–S5.

### 2.3 Intermolecular Interactions by Hirshfeld Surfaces Methodology

Hirshfeld’s surfaces^23,24^ and two-dimensional (2D) fingerprint plots^25,26^ were obtained using CrystalExplorer 17.5 software to examine the crystal intermolecular contacts between the different chemical species present. The CIF file used has been deposited into the Cambridge Structural Database (CSD) under code 2372426 (300 K). Hirshfeld’s surfaces were mapped using the normalized distance property (*d*_norm_), which is defined as the distance from a specific point on the surface to the nearest external atom (*d*_e_) and the nearest internal atom (*d*_i_), both normalized by the van der Waals radius. Furthermore, through the same computational calculations, the shape index and curvature of the molecular unit were generated to perform a topological analysis of the contacts. 2D fingerprint plots were displayed as a function of the distance from a specific point on the Hirshfeld’s surface in terms of *d*_e_ and *d*_i_.^27^ The generated patterns encompassed all intermolecular interactions, enabling the quantification of specific contacts.

### 2.4 Ultraviolet–Visible (UV–Vis) Spectroscopy Methodology

Absorbance spectra were obtained using a dual-beam UV–vis spectrophotometer (Evolution model, Waltham, MO, USA) equipped with a deuterium lamp operating over 200–900 nm. Measurements were performed in quartz cuvettes with an optical path of 0.1 cm. 20 mg of the [Cu­(l-Ile)­(phen)­(H_2_O)]­Cl·2H_2_O complex powder was dissolved in 5 mL of water. For UV–vis measurements, the complex was dissolved in water and further diluted to keep absorbance below the instrument’s linear range (A ≤ 1.5) using a 0.10 cm quartz cuvette.

### 2.5 Vibrational Analyses Methodology

The infrared (IR) spectrum of the complex was measured in the 4000–400 cm^–1^ (mid-infrared) range in attenuated total reflectance (ATR) mode using a Bruker Vertex 70v spectrometer (Billerica, MA). The A225/Q Platinum ATR accessory, equipped with a wideband RT-DLa TGS detector (MIR–FIR) with a 6 mm aperture, was used for measurements. Spectra were recorded with a resolution of 4 cm^–1^ and averaged over 100 scans.

The Raman spectrum was obtained in the region of 40–3400 cm^–1^ using a LabRAM HR Evolution spectrometer (Horiba, Ann Arbor, MI, USA) equipped with a CCD (charge-coupled device) detector thermoelectrostatically cooled by a Peltier system. Excitation was performed with a green solid-state laser (λ = 514 nm), with an approximate power of 2.0 mW. The spectrum was recorded with a spectral resolution of 4 cm^–1^, totaling four accumulations and an acquisition time of 90 s.

### 2.6 Molecular Docking Methodology

For the molecular docking studies, the biologically relevant cationic species [Cu­(l-Ile)­(phen)­(H_2_O)]^+^ was used as the ligand. The chloride counterion and lattice water molecules present in the crystalline structure were excluded, as they are not expected to remain associated with the complex under physiological conditions. The cationic complex was energy-minimized and prepared using AutoDock Tools software (version 1.5.7).^28^ DNA (PDB ID: 1BNA) and bovine serum albumin (BSA) (PDB ID: 4F5S) structures were obtained from the Protein Data Bank and prepared by removing the free molecules and heteroatoms, followed by the addition of polar hydrogens and Kollman charges.^29,30^ The protein structure was prepared by excluding heteroatoms and water molecules and then adding Kollman charges and polar hydrogens. The PDBQT files of the macromolecules and complexes were generated accordingly. The files autodock4.exe, autogrId4.exe, and AD4.1_bound.exe were separated in a folder together with the. PDB files of DNA, BSA, and the [Cu­(l-Ile)­(phen)­(H_2_O)]^+^ complex. The docking results were then visualized and analyzed by using Discovery Studio software.^31^

### 2.7 Antitumor Activity Assay in SiHa Cells and Fibroblasts

For the cytotoxicity assays, two cell lines were used: SiHa (HTB-35, ATCC, Manassas, VA), derived from human cervical epithelial carcinoma, and GM07492A (CVCL_7467, NIGMS), a nontumor human lung fibroblast cell line used as a normal control.

SiHa and GM07492A cells were seeded in 96-well plates at densities of 1 × 10^4^ cells/well (SiHa) and 2 × 10^4^ cells/well (GM07492A) in Dulbecco’s modified Eagle’s medium (DMEM; Gibco, Waltham, MA) supplemented with 10% fetal bovine serum (FBS; Gibco, Paisley, U.K.), amphotericin B (250 μg/mL), and penicillin/streptomycin (10,000 μg/mL). Cells were incubated at 37 °C in a humidified atmosphere containing 5% CO_2_ for 24 h to allow adhesion

The [Cu­(l-Ile)­(phen)­(H_2_O)]­Cl·2H_2_O complex was sterilized under UV light for 15 min, dissolved in dimethyl sulfoxide (DMSO; Sigma-Aldrich, São Paulo, Brazil) to obtain a 5 mM stock solution, and homogenized for 30 s. After the adhesion period, the culture medium was replaced, and cells were treated with serial dilutions of the complex (50–0.195 μM) for 72 h. Cisplatin (Sigma-Aldrich, St. Louis, MO) was used as a positive control (initial concentration from 666.0 to 5.2 μM) and tested under the same experimental conditions. Cells treated with culture medium alone served as a negative control.

Cell viability was determined using the MTT assay^32^ as previously described.^3^ After treatment, the medium was replaced with 90 μL of fresh culture medium plus 10 μL of MTT solution (0.5 mg/mL), followed by incubation for 3 h to allow formazan formation. The supernatant was discarded, crystals were solubilized in 100 μL of DMSO, and absorbance was measured at 540 nm using a microplate reader (BioTek ELx808, BioTek Industries, Highland Park, USA).

Results were expressed as a percentage of cell death for SiHa cells and a percentage of cell viability for GM07492A cells relative to the negative control. The selectivity index (SI) was calculated to compare the sensitivities of the normal and tumor cell lines using eq 1.

1 $$SI=\frac{{IC}_{50}\;\mathrm{GM}07492A}{{IC}_{50}\;SiHa}$$

where IC_50_ refers to the half maximal inhibitory concentration. SI > 1 indicates that the [Cu­(l-Ile)­(phen)­(H_2_O)]­Cl·2H_2_O complex has selectivity against cancer cells.

### 2.8 Statistical Analysis Methodology

The data were analyzed using a one-way parametric ANOVA, followed by Tukey’s post hoc test. The results are presented as mean ± standard deviation (SD) and represent two independent experiments. Significant differences from the control group (Medium) were indicated by *p < 0.05 and ^#^ *p* < 0.0001.

## 3 Results and Discussion

### 3.1 Single-Crystal X-ray Diffraction and Structure Determination of [Cu­(l-Ile)­(phen)­(H_2_O)]­Cl·2H_2_O Crystal

The complex was structurally characterized using a SCXRD technique under ambient conditions (300 K), and the resulting diffraction pattern is shown in Figure 1a. Selected crystallographic data are summarized in Table 1. Molecular structure and unit cell of the complex are shown in Figure 1b,c, the crystallographic structure of the complex exhibits a monoclinic system and *P*2_1_ (C_2_^2^) space group, with 2 formulas per unit cell (*Z* = 2) and the following lattice parameters *a* = 11.852(5), *b* = 6.907(3), and *c* = 13.888(7) Å, with angle β = 108.129(2)° and volume *V* = 1080.41(9) Å^3^. The Cu­(II) cation exhibits a distorted square pyramidal geometry, in which the metallic ion coordinates with two phen ligand nitrogen atoms, one amino nitrogen atom, one carboxylate oxygen atom of l-Ile at equatorial positions, and one water oxygen atom at axial positions. Selected bond lengths and angles are given in Tables 2 and 3, respectively.

**Figure 1:**
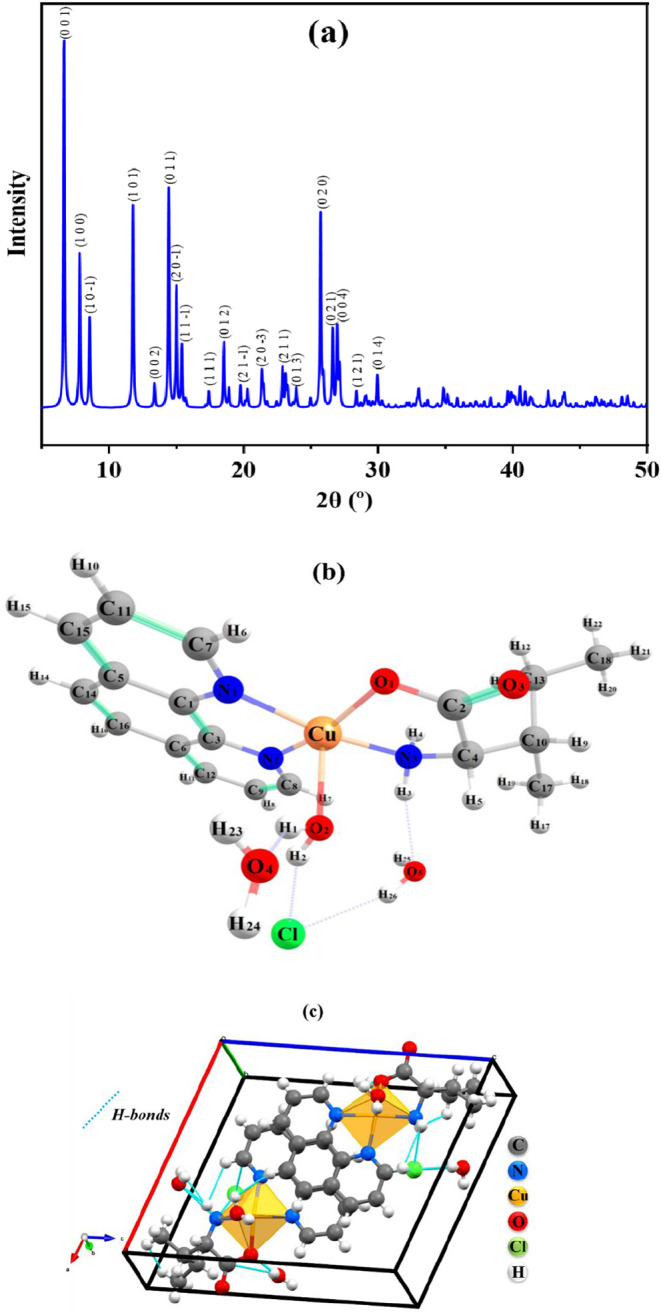
(a) SCXRD pattern for the single crystal of [Cu­(l-Ile)­(phen)­(H_2_O)]­Cl·H_2_O complex at 300 K, (b) Molecular structure of the complex at molecular level and (c) unit cell of the monoclinic system in the *abc* plane, highlighting some intermolecular interactions formed between the coordination complex, involving Cl^–^ ions and H_2_O molecules.

**Table 1:** Crystal Data and Structure Refinement for [Cu­(l-Ile)­(phen)­(H_2_O)]­Cl·2H_2_O Complex^a^

	[Cu(l-Ile)(phen)(H_2_O)]Cl·2H_2_O
empirical formula	C_18_H_26_ClCuN_3_O_5_
formula weight	463.41
temperature (K)	300
crystal system	monoclinic
space group	*P*2_1_
*a* (Å)	11.8521(5)
*b* (Å)	6.9068(3)
*c* (Å)	13.8877(7)
α (deg)	90
β (deg)	108.129(2)
γ (deg)	90
volume (Å^3^)	1080.41(9)
*Z*	2
ρ_calc_ (g/cm^3^)	1.424
μ (mm^–1^)	1.167
*F*(000)	482.0
crystal size (mm^3^)	0.231 × 0.208 × 0.135
radiation	Mo Kα (λ = 0.71073)
2θ range for data collection (deg)	6.108–55.014
index ranges	–15 ≤ *h* ≤ 15, –8 ≤ *k* ≤ 8, –18 ≤ *l* ≤ 18
reflections collected	16,551
independent reflections	4903 [*R*_int_ = 0.0312, *R*_sigma_ = 0.0356]
data/restraints/parameters	4903/1/263
goodness-of-fit on *F* ^2^	1.024
final *R* indexes [*I* ≥ 2σ(*I*)]	*R*_1_ = 0.0318, w*R*_2_ = 0.0845
final *R* indexes [all data]	*R*_1_ = 0.0368, w*R*_2_ = 0.0885
Flack parameter	0.025(18)

a The supplementary crystallographic data for this work are found under the code CCDC 2372426 and can be accessed/obtained free of charge from the CSD through the link: https://bdec.dotlib.com.br/inicio_asm/application/webcsd.

**Table 2:** Bond Lengths for [Cu­(l-Ile)­(phen)­(H_2_O)]­Cl·2H_2_O Complex

atom	length (Å)	atom	length (Å)
Cu1–O1	1.944(2)	C5–C4	1.409(4)
Cu1–O4	2.222(3)	C14–C15	1.541(5)
Cu1–N2	2.004(2)	C7–C8	1.438(5)
Cu1–N1	2.018(2)	C7–C10	1.390(6)
Cu1–N3	1.982(3)	C4–C3	1.408(6)
O1–C13	1.276(4)	C4–C9	1.416(6)
N2–C6	1.365(4)	C12–C11	1.388(5)
N2–C12	1.326(4)	C1–C2	1.411(5)
N1–C5	1.356(4)	C2–C3	1.343(6)
N1–C1	1.330(4)	C15–C17	1.518(8)
O2–C13	1.224(4)	C15–C16	1.523(7)
N3–C14	1.475(4)	C11–C10	1.370(6)
C6–C5	1.416(5)	C17–C18	1.528(7)
C6–C7	1.395(4)	C8–C9	1.342(7)
C13–C14	1.526(5)		

**Table 3:** Bond Angles for [Cu­(l-Ile)­(phen)­(H_2_O)]­Cl·2H_2_O Complex

atom	angle (deg)	Atom	angle (deg)
O1–Cu–O4	92.7(11)	N1–C5–C6	116.9(2)
O1–Cu–N2	93.2(11)	N1–C5–C4	122.9(3)
O1–Cu–N1	166.5(14)	C4–C5–C6	120.2(3)
O1–Cu–N3	83.4(11)	N3–C14–C13	109.4(3)
N2–Cu–O4	99.7(14)	N3–C14–C15	113.7(3)
N2–Cu–N1	82.0(10)	C13–C14–C15	112.9(3)
N1–Cu–O4	100.5(15)	C6–C7–C8	117.6(4)
N3–Cu–O4	91.0(12)	C10–C7–C6	117.3(3)
N3–Cu–N2	168.9(15)	C10–C7–C8	125.1(3)
N3–Cu–N1	98.9(11)	C5–C4–C9	118.5(4)
C13–O1–Cu	114.0(2)	C3–C4–C5	116.5(3)
C6–N2–Cu	112.5(2)	C3–C4–C9	125.0(3)
C12–N2–Cu	129.4(2)	N2–C12–C11	122.2(3)
C12–N2–C6	118.1(3)	N1–C1–C2	121.7(4)
C5–N1–Cu	112.1(2)	C3–C2–C1	119.8(4)
C1–N1–Cu	129.2(2)	C17–C15–C14	112.0(4)
C1–N1–C5	118.6(3)	C17–C15–C16	114.0(5)
C14–N3–Cu	108.1(2)	C16–C15–C14	110.1(4)
N2–C6–C5	116.4(3)	C10–C11–C12	119.9(4)
N2–C6–C7	122.9(3)	C2–C3–C4	120.5(3)
C7–C6–C5	120.6(3)	C15–C17–C18	113.6(5)
O1–C13–C14	117.0(3)	C9–C8–C7	122.0(3)
O2–C13–O1	122.7(4)	C11–C10–C7	119.6(3)
O2–C13–C14	120.3(3)	C8–C9–C4	121.1(3)

A ternary Cu­(II) complex containing L-Ille and phen, with nitrate as the counterion, was previously reported by Chen et al.^17^ While both compounds exhibit a comparable primary coordination environment around the Cu­(II) center, the present structure differs in the nature of the counterion: chloride rather than nitrate. This difference is reflected in the crystallographic formulation and contents of the asymmetric unit, although the metal coordination geometry remains essentially preserved. Importantly, although the primary coordination environment is preserved, the substitution of nitrate by chloride results in distinct hydrogen-bonding motifs and intermolecular connectivity. These differences are quantitatively reflected in contributions and directly affect the solid’s supramolecular organization, highlighting the noninnocent role of the counterion in crystal engineering.

Equatorial bond lengths were Cu–N1 = 2.018(2) Å, Cu–N2 = 2.004(2) Å, Cu–N3 = 1.982(3) Å, Cu–O1 = 1.944(2) Å and weakly bonded axial bond distance were Cu–O4 = 2.222(3) Å. The N–Cu–N, N–Cu–O, and O–Cu–O bond angles ranged from 81.98(10) to 168.88(15)^o^. The crystal structure is mainly secured by hydrogen bonds: N–H···O, O–H···O, and O–H···Cl (Table S3). It should be noted that, as a hydrogen bond donor, the amine group (−NH_2_) of l-Ile interacts with the oxygen atom of an uncoordinated water molecule. On the other hand, the oxygen of the carboxyl group of water in an axial position acts as both hydrogen bond donor and acceptor, participating in two hydrogen bond interactions, namely, one with the hydrogen of the uncoordinated water and the other with the uncoordinated chlorine atom, thereby ensuring the stability of the crystal structure.

In the refined SCXRD model, the chloride ion is located outside the Cu­(II) primary coordination sphere and was identified from the electron-density map and refined with full occupancy as a counterion. Two water molecules are present: one coordinated axially to Cu­(II) and one lattice water molecule. Their positions and orientations are supported by refined O–Cu/O–H geometries and by the hydrogen-bond lattice summarized in Table S3, which rationalizes the crystal packing stability.

### 3.2 Analysis of Intermolecular Interactions by Hirshfeld Surfaces

The Hirshfeld surface is a technique that allows visualization and analysis of intermolecular interactions in crystals, providing a 3D representation of the electron density of a molecule in a crystal. This surface is used to quantify the interactions between neighboring molecules, helping identify contact areas and the nature of these interactions, such as van der Waals forces, hydrogen bonds, and electrostatic interactions.^24^ Hirshfeld surface mapping and fingerprint plot interpretation were conducted by following established protocols.^23–27^

In this study, the analysis of the Hirshfeld surface revealed important interactions that stabilize the crystal structure. Through mappings, such as *d*_i_, *d*_e_, *d*_norm_, and curvature, specific regions on the surface of a molecule that interacts with other molecules in the crystal are identified.^27^ Figure 2a shows a combined mapping of the *d*_i_ and *d*_e_ distances, helping identify regions of intermolecular contact and their respective intensities. Regions with significant interactions are highlighted in colors, ranging from blue to red, where blue represents weak interactions, and red represents strong interactions in the [Cu­(l-Ile)­(phen)­(H_2_O)]­Cl·2H_2_O complex, represented by the O, H, and Cl atoms. Figure 2b represents the distance between point of interest in surface and nucleus of the closest atom inside molecules in the [Cu­(l-Ile)­(phen)­(H_2_O)]­Cl·2H_2_O complex. Figure 2c shows the distance between the point of interest in the surface and the closest atom outside molecules in the complex. Figure 2d shows the curvature of the surface and reveals the areas where noncovalent interactions occur, such as π–π stacking or van der Waals interactions. Flat regions in green represent low curvature values, while the blue contours at the edges show high curvature values in the complex. Figure 2e is a useful tool to identify flat regions of the surface that participate in π–π interactions. These interactions occur between the aromatic rings of the [Cu­(l-Ile)­(phen)­(H_2_O)]­Cl·2H_2_O complex, such as the rings of phen. These regions of interaction, represented by triangles, suggest an overlap of the π electron clouds, which contributes to the stabilization of the crystal structure through π–π interactions between adjacent molecules. Figure 2f shows the molecular fragments involved in the interactions, allowing clearer visualization of the specific interactions between different parts of the molecule and its neighbors in the [Cu­(l-Ile)­(phen)­(H_2_O)]­Cl·2H_2_O complex.

**Figure 2:**
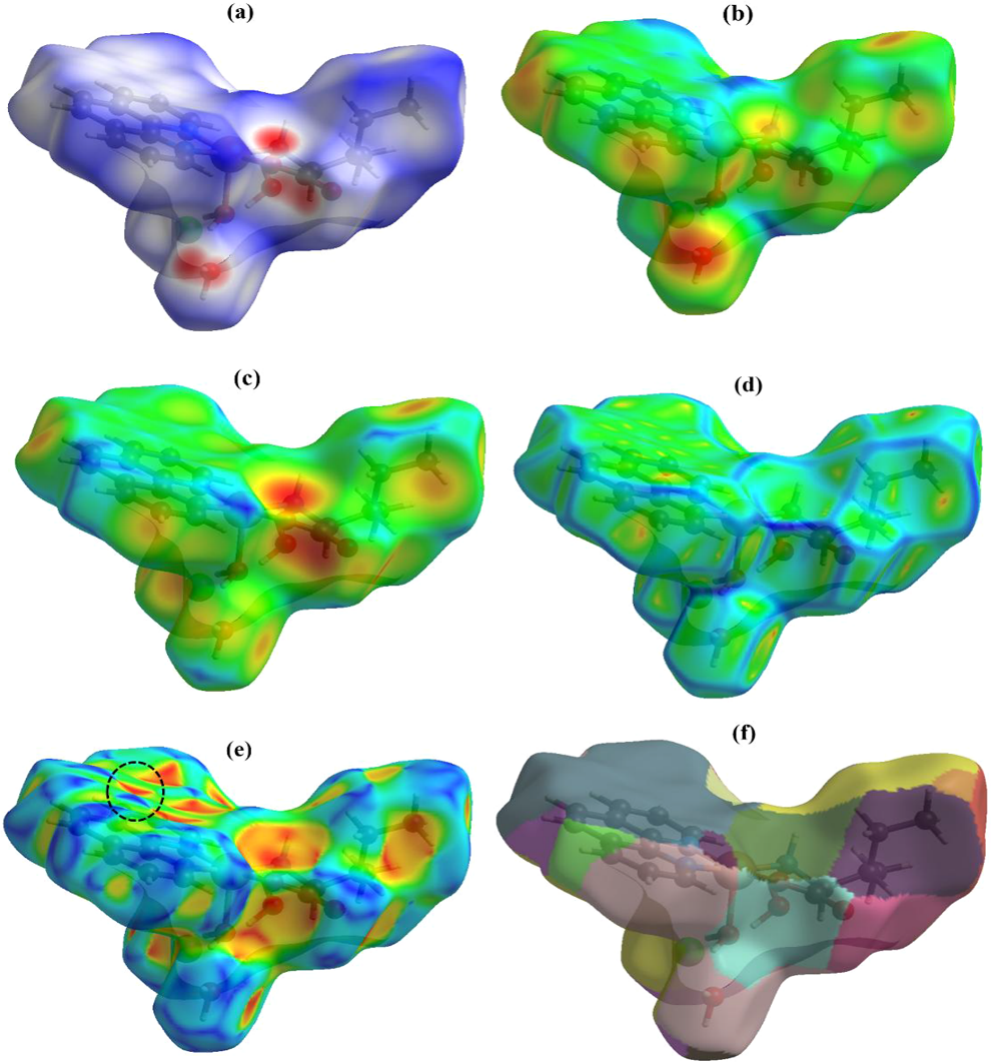
Hirshfeld surface of the [Cu­(l-Ile)­(phen)­(H_2_O)]­Cl·2H_2_O crystal molecule mapped according to (a) *d*_norm_, (b) *d*_i_, (c) *d*_e_, and (d) curvature, (e) shape index, and (f) fragments.

As mentioned, the chlorine atom in the complex acts as a counterion and is not directly coordinated to the Cu­(II) metal center. Its influence occurs mainly through electrostatic interactions with the complex. Uncoordinated water molecules form hydrogen bonds with other molecules, thereby enhancing the crystal’s structural stability. These water molecules can also influence the formation of the crystal lattice by interacting with polar groups in the complex via hydrogen bonds.

The 2D fingerprint plots,^25,26^ as shown in Figure 3, are a detailed visual representation of intermolecular interactions on the Hirshfeld surface of a molecule in the crystal. These plots provide quantitative information about the distances between atoms in adjacent molecules, highlighting the interactions within the crystal.

**Figure 3:**
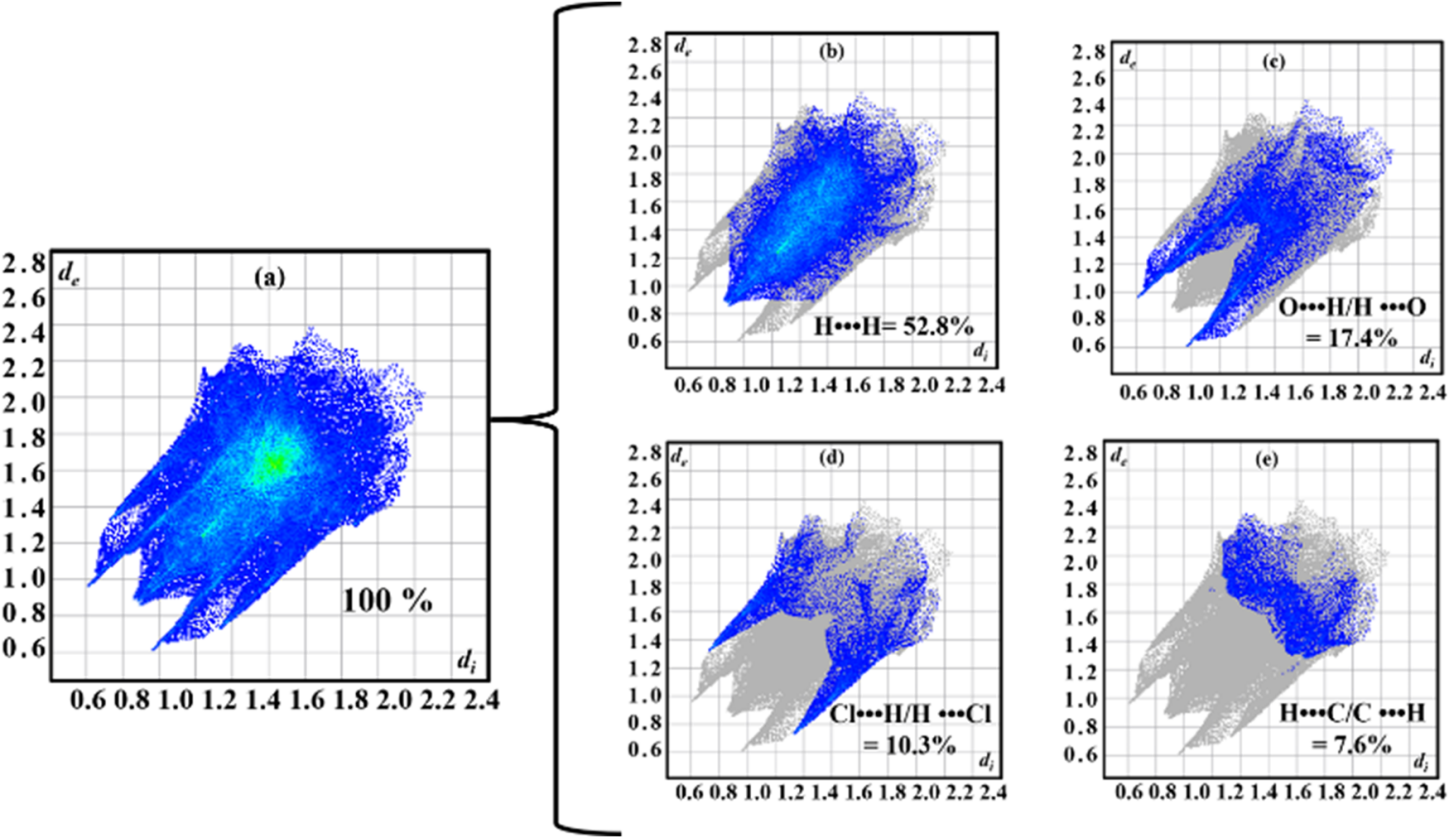
(a) Full 2D fingerprint plot of the [Cu­(l-Ile)­(phen)­(H_2_O)]­Cl·2H_2_O crystal and specific fingerprint graphs of key interactions: (b) H···H, (c) O···H/H···O, (d) Cl···H/H···Cl, and (e) H···C/C···H.

The complete graph provides an overview of all interactions occurring in the crystal (Figure 3a). Specific graphs focus on each type of interaction (Figure 3b–e), making it easier to identify which interactions are most significant for crystal stabilization. Each 2D graph represents the contribution of a specific interaction type, making it easier to identify which interactions are the most significant for crystal stabilization. The graphs are constructed with the *d*_*i*_ (inner distance) axis on the *x*-axis and *d*_e_ (outer distance) on the *y*-axis. Each point on the graph corresponds to an interaction between two atoms of neighboring molecules, and its location indicates the distance between the two atoms.

As observed, the most important interactions are those between H···H (52.8%), O···H/H···O (17.4%), and Cl···H/H···Cl (10.3%), shown in Figure 3b–d, according to the representation on the Hirshfeld surface concerning the *d*_norm_. Additionally, a relevant contribution of the H···C/C···H interactions (7.6%) (Figure 3e) can be noted, related to the aromatic rings of phen. The remaining percentages are associated with less significant interactions within the structure.

The patterns formed by the points on the graph reveal the nature of the interactions. For example, the H···H interactions shown in Figure 3b represent van der Waals interactions, which are generally weak but predominate due to the abundance of hydrogen atoms. The O···H/H···O hydrogen-bonding interactions, as shown in Figure 3c, appear as clusters of points in specific areas, highlighting the strong attraction between hydrogen and oxygen. In addition, elongated peaks appear near the axes, as in the Cl···H/H···Cl interactions shown in Figure 3d, reflecting the prevalence of these interactions at the contact surfaces that contribute to the crystal’s structural stability. H···H interactions generally make the largest contribution due to the abundance of hydrogen atoms. Still, interactions, such as O···H/H···O or Cl···H/H···Cl, although less frequent, can play a crucial role in hydrogen bond formation or in electrostatic stabilization.

In this way, the graphs, together with the Hirshfeld surface analysis, provide deep insight into the molecular forces that hold the crystal structure together, revealing the importance of each interaction. These specific interaction graphs help unravel how the different fragments of the molecule (such as the uncoordinated chlorine and water and the aromatic rings of phen) contribute to the formation and stability of the crystal. Thus, graphical analysis enables the identification of which interactions predominate and how these intermolecular forces influence the final crystal architecture. Furthermore, these graphs are fundamental to understanding the van der Waals interactions, hydrogen bonds, and electrostatic interactions that occur in the crystal structure, providing a deeper understanding of the 3D organization of the complexes.

A comparison with the structurally related Cu­(II)–L-Ille–phen complex containing nitrate as counterion, reported by Chen et al.,^17^ highlights the role of the counterion in shaping the hydrogen-bonding lattice. In both structures, the counterions interact with coordinated and lattice water molecules, thereby stabilizing the crystal packing. However, the nature of these interactions varies with the counterion. In the nitrate-containing complex, hydrogen bonding occurs predominantly through O–H···O interactions involving the oxygen atoms of the nitrate anion. In contrast, in the present chloride-containing complex, the chloride ion acts exclusively as a hydrogen-bond acceptor in O–H···Cl interactions mediated by water molecules. These differences lead to distinct intermolecular connectivity patterns, as reflected in the Hirshfeld surface maps and the relative contributions in the 2D fingerprint plots.

Figure 4 presents a detailed model of the interactions relevant to the molecule’s crystal structure. The blue dotted lines represent the contacts associated with the bonds. Thus, these results confirm the information presented in Figure 3b–e, indicating that the primary interactions between the molecules are mainly due to hydrogen bonds.

**Figure 4:**
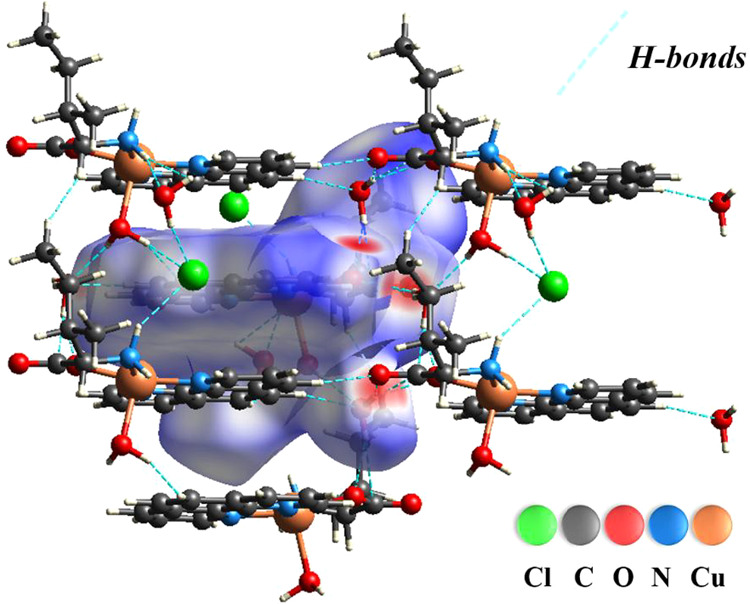
Relevant intermolecular interactions in the crystal of the [Cu­(l-Ile)­(phen)­(H_2_O)]­Cl·2H_2_O complex: blue dashed lines (H···H, O···H/H···O, Cl···H/H···Cl, and H···C/C···H).

Overall, the Hirshfeld surface analysis demonstrates that the solid-state architecture of the complex is governed by a cooperative lattice of hydrogen-bonding and weak dispersive interactions, in which the chloride counterion and water molecules play a key structural role. These supramolecular features are not only crystallographic details but are also relevant to understanding solid-state stability and potential dissolution behavior in biological environments.

It should be emphasized that the quantitative contribution of H···H, O···H/H···O, and Cl···H/H···Cl interactions is not merely descriptive but reflects the cooperative nature of the supramolecular stabilization in the solid state. Similar interaction patterns have been reported for Cu­(II)-phen systems^4,7,8^ and are known to influence crystal cohesion, hydration behavior, and solid-state robustness, which are relevant parameters for pharmaceutical handling and formulation.

### 3.3 UV–Vis Spectroscopy

UV–vis spectroscopy is a fundamental tool for investigating electronic transitions in metal–organic complexes, especially those containing Cu­(II) ions. These transitions provide valuable information about the electronic environment and the coordination geometry of the metal center.^2,3^ In the case of the [Cu­(l-Ile)­(phen)­(H_2_O)]­Cl·2H_2_O complex, the absorption spectrum was obtained in aqueous medium and is shown in Figure 5. The Cu­(II) (d^9^) ion presents a set of *d*–*d* electronic transitions, which are spin-allowed but symmetry-forbidden (Laporte’s rule),^32^ which generally results in low-intensity bands. However, due to the distorted square pyramidal geometry of the complex, the ideal symmetry is broken and the prohibition is relaxed, allowing the observation of these bands. In the spectrum of the complex, a broad and low-intensity band is observed in the region of 60–750 nm, attributed to the *d*–*d* transitions of Cu­(II), involving excitations between d orbitals of different energies (for example, transitions from the *d*_*x* ^2^–*y* ^2^_ orbital to the *d*_*xz*_, *d*_*yz*_, or *d*_*z* ^2^_). The distortion of the geometry around the metal center leads to the energetic separation of these orbitals and, therefore, to the occurrence of multiple overlapping transitions. In addition to the *d*–*d* transitions, the spectrum exhibits intense bands in the 250–350 nm region that are typical of ligand-to-metal charge transfer. These transitions involve the transfer of electrons from the π orbitals of the aromatic ligand phen or the oxygenated groups of l-Ile to the empty orbitals of Cu­(II), especially the *d*_*x* ^2^–*y* ^2^_ orbital, which is higher in energy in the ligand field. The simultaneous presence of *d*–*d* and ligand-to-metal charge transfer bands is compatible with the pentacoordinate (square pyramidal) geometry of the complex, as also corroborated by crystallographic data (see inset in Figure 5). These spectroscopic data confirm the formation of the proposed complex and provide insights into its electronic properties, which are related to its biological activity.

**Figure 5:**
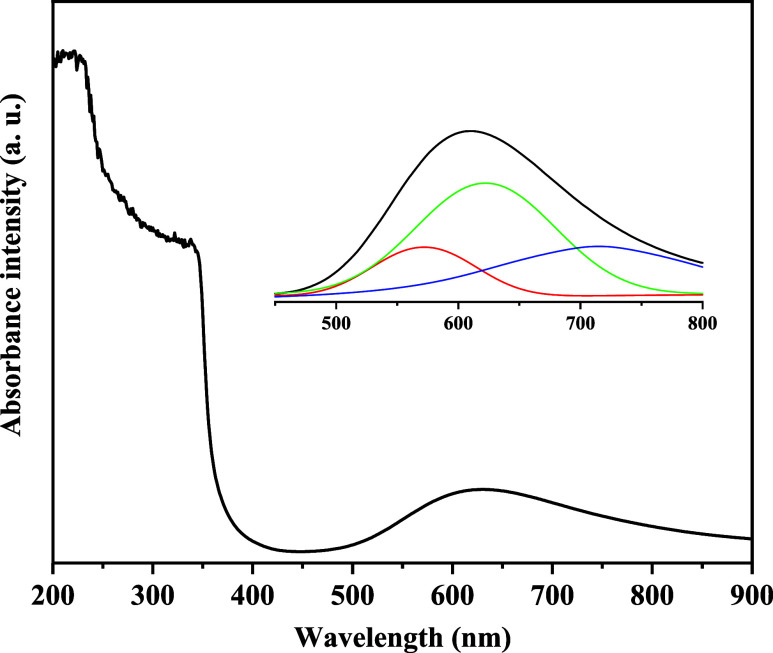
Optical absorption spectrum of [Cu­(l-Ile)­(phen)­(H_2_O)]­Cl·2H_2_O crystal solution in aqueous medium in the 200–900 nm region. The inset shows the absorption spectrum in the 450–800 nm region, with three smaller bands corresponding to *d-d* transitions.

The spectroscopic features observed herein are fully consistent with the distorted square-pyramidal geometry determined crystallographically and provide complementary evidence for the electronic configuration of the Cu­(II) center, which is directly relevant to its redox behavior and potential biological reactivity.^3,7^

### 3.4 Vibrational Analyses

FT-IR and Raman spectroscopy identified essential functional groups in the powder crystal of the [Cu­(l-Ile)­(phen)­(H_2_O)]­Cl·2H_2_O complex. The IR absorption spectrum is presented in Figure 6a, and the Raman spectrum is shown in Figure 6b. Table 4 summarizes the main bands observed and their spectral assignments compared to values described in the literature.

**Figure 6:**
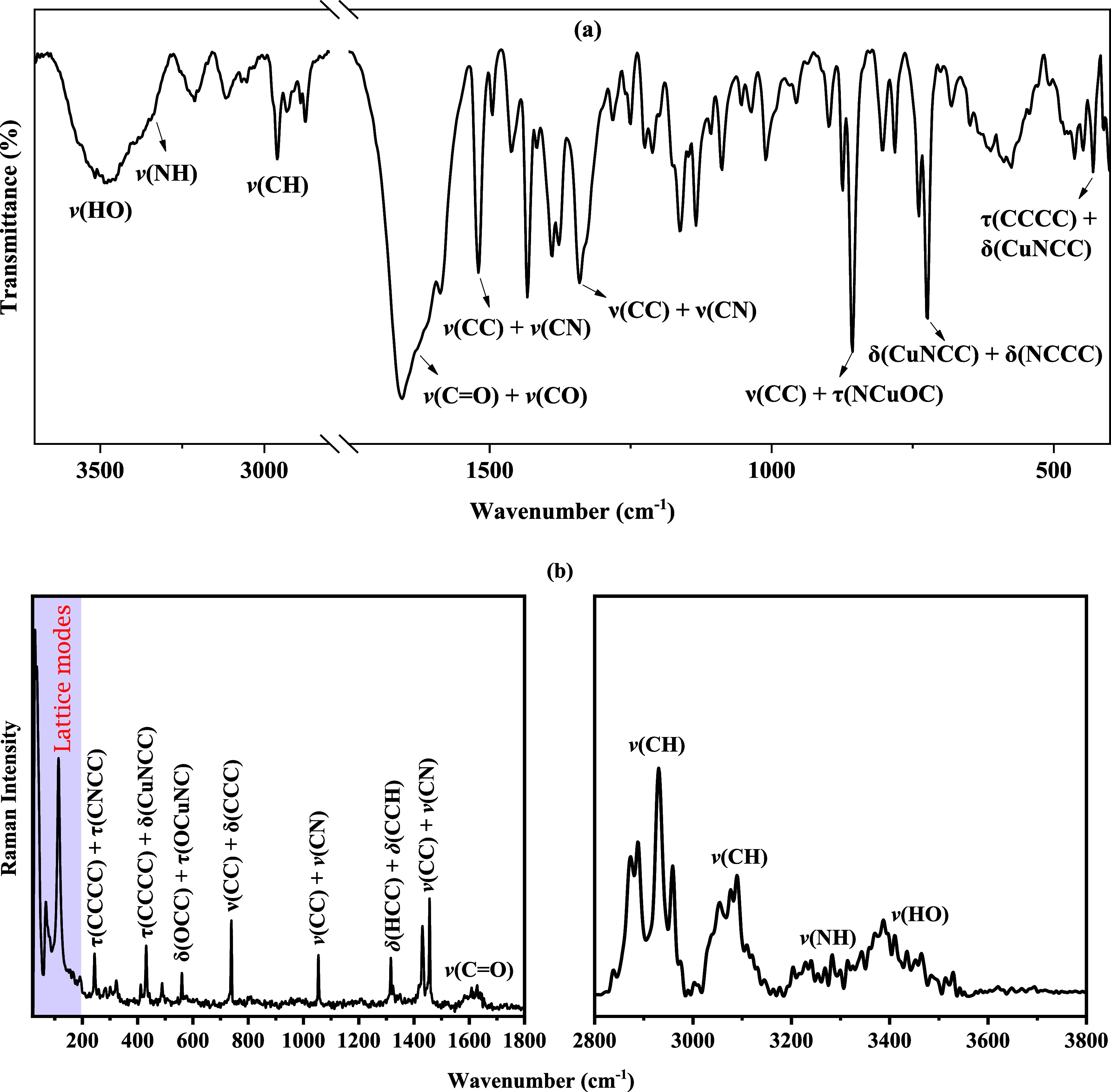
Experimental (a) IR and (b) Raman spectra of the [Cu­(l-Ile)­(phen)­(H_2_O)]­Cl·2H_2_O crystal.

**Table 4:** Experimental Values of IR and Raman Wavenumbers, and with Vibrational Mode Assignments for the [Cu­(l-Ile)­(phen)­(H_2_O)]­Cl·2H_2_O Complex^a^

ω_IR_	ω_Raman_	assignments
3572		ν(H–O)^2,7^
3477	3462	ν(H–O)^2,7^
3248		ν(N–H_2_)^6^
3230	3232	ν(N–H_2_)^2,3,33^
3213		ν(C–H)^3,6,33,34^
3199	3205	ν(C–H)^3,6,33,34^
3188		ν(C–H)^3,6,33,34^
3127		ν(C–H)^3,6,33,34^
3117		ν(C–H)^3,6,33,34^
3098	3090	ν(C–H)^3,6,33,34^
3068	3075	ν(C–H)^3,6,33,34^
3052	3054	ν(C–H)^3,6,33,34^
2961	2959	ν(C–H)^3,6,33,34^
2932	2931	ν(C–H)^3,6,33,34^
2914		ν(C–H)^3,6,33,34^
2890	2889	ν(C–H)^3,6,33,34^
2874	2872	ν(C–H)^3,6,33,34^
1658		ν(C–C) + ν(C–N)^2,3,33^
1635	1639	ν(C = O) + ν(C–O)^2,3,33^
1613	1624	ν(C–C) + ν(C–N)^2,3,33^
1588	1606	ν(C–C) + ν(C–N)^2,3,33^
1580	1585	ν(C–C) + ν(C–N)^2,3,33^
1520	1504	ν(C–C) + ν(C–N)^2,3,33^
1495		ν(C–C) + ν(C–N)^2,3,33^
1460	1454	ν(C–C) + ν(C–N)^2,3,33^
	1444	δ(H–C–C) + δ(C–C–H)^2,3^
1433	1429	ν(C–O) + δ(N–C–H)^2,3^
1391		ν(C–N) + δ(N–C–H)^2,3^
1377		δ(H–C–H) + δ(H–C–H)^2,3^
1340	1348	ν(C–C) + ν(C–N)^2,3,33^
1326	1323	δ(C–C–H) + δ(H–C–C)^2,3^
	1314	δ(H–C–C) + δ(C–C–H)^2,3,33^
1282	1298	ν(C–N) + δ(N–C–H)^2,3,33^
1251		ν(O–C) + δ(H–C–C)^2,3,33^
1225		ν(N–C) + δ(N–C–H)^2,3^
1212		ν(O–C) + δ(H–C–C)^2,3^
1201		ν(C–C)^2,3,33^
1177		ν(C–C) *+* δ(C–C–C)^2,3^
1162		ν(C–C) *+* δ(C–C–C)^2,3^
1133		ν(C–C) *+* δ(C–C–C)^2,3^
1108		ν(C–C) + ν(C–N)^2,3^
1089		δ(C–N–C) + δ(C–C–C)^2,3^
1055	1052	ν(C–C) + ν(C–N)^2,3,33^
1036		ν(C–C)^2,3,33^
1010		ν(C–C)^2,3,33^
994		ν(C–C)^2,3,33^
968		ν(C–C) + ν(C–N)^2,3,33^
955		ν(C–C) + δ(C–C–C)^2,3,33^
897		ν(O–C) + ν(C–C) + τ(Cu–N–C–C)^2,3,33^
874		ν(O–C) + ν(C–C)^2,3,33^
855	824	ν(C–C) + τ(N–Cu–O–C)^2,3,33^
802	802	δ(O–C = O)^2,3,33^
782		δ(O–C–O) + ν(O–C)^2,3^
739	736	ν(C–C) + δ(C–C–C)^2,3,33^
724	720	δ(Cu–N–C–C) + δ(N–C–C–C)^2,3,33^
701		δ(C–C–C) + ν(C–C)^2,3^
648		ν(C–C) + ν(C–N)^2,3,33^
633		τ(C–C–C–C) + δ(N–C–C)^2,3,33^
619		τ(N–Cu–O–C) + ν(Cu–N) + δ(Cu–N–H)^2,3,33^
610		δ(C–N–C) + δ(C–C–C)^2,3,33^
592	602	ν(C–C) + δ(C–C–C)^2,3,33^
576	575	δ(O–C–C) + τ(O–Cu–N–C)^2,3,33^
560		τ(Cu–N–C–C)^2,3,33^
543		δ(O–C–C)^2,3,33^
528		δ(C–C–C)^2,3,33^
507		δ(C–C–C) + ν(N–C)^2,3,33^
488	485	δ(C–C–C) + ν(N–C)^2,3,33^
480		δ(C–C–C) + ν(N–C)^2,3,33^
473		δ(C–C–C) + ν(Cu–N)^2,3,33^
463	466	δ(C–C–C) + ν(C–C)^2,3,33^
448		ν(C–C) + δ(C–C–C)^2,3,33^
430	427	τ(C–C–C–C) + δ(Cu–N–C–C)^2,3,33^
411		ν(C–C) + δ(C–C–C)^2,3,33^
401	406	ν(C–C) + δ(C–C–C)^2,3,33^
	319	ν(Cu–O) + δ(O–C = O)^2,3,33^
	299	δ(Cu–N–C)^2,3,33^
	276	ν(Cu–N) + ν(C–C)^2,3,33^
	253	τ(Cu–N–C–C) + τ(N–C–C–C)^2,3,33^
	241	τ(C–C–C–C) + τ(C–N–C–C)^2,3^
	185	lattice modes
	166	
	146	
	109	
	64	
	28	

a Nomenclature: τ = torsion; δ = bending; ν = stretching.

Bands observed in the spectral region between 3477 and 3572 cm^–1^ of IR spectrum are related to the stretching’s of O–H bonds due to the coordination and crystallization of water molecules.^2,7^ This attribution is confirmed by the presence of a related band at about 3462 cm^–1^ in the Raman spectrum. Vibrations attributed to the stretching of N–H bonds from the amine group appear in the IR spectrum at about 3248 and 3230 cm^–1^, and with a corresponding band at 3232 cm^–1^ in the Raman, confirming the presence of primary amines bound to the metal center.^33^

In the spectral range from 3213 to 2874 cm^–1^, multiple bands attributed to C–H stretching were observed in both IR and Raman spectra. This vibrational feature reveals the complexity of the chemical environment related to the alkyl groups present in the l-Ile and phen ligands, and is characterized by intense and well-defined bands, such as those around 3098/3090 cm^–1^, 2961/2959 cm^–1^, and 2874/2872 cm^–1^ (IR/Raman).

The region between 1580 and 1658 cm^–1^ contains significant bands attributed to mixed vibrational modes involving stretching of C=C, C=N, C=O, and C–O bonds, reflecting the overlapping vibrations of the carbonyl and amide groups.^2^ Bands at about 1580 and 1635 cm^–1^ (IR), and the bands centered at 1585, 1606, and 1639 cm^–1^ (Raman), indicate active involvement of these groups in coordinating the cupric ion. This band is crucial for confirming the coordination of electron-donating groups to the metal center, especially involving the oxygen and nitrogen atmos.

Vibration bands associated with stretching of C–N and C–C bonds, and deformations, are widely distributed between 1000 and 1495 cm^–1^. The presence of bands, such as 1340/1348 cm^–1^ and 1282/1298 cm^–1^, in both techniques (IR/Raman) highlights the active participation of these modes in different symmetries and structural contexts.^2^ This band also reflects the ligands’ mixed structure, with aliphatic (l-Ile) and aromatic (phen) characteristics.

Particular attention should be paid to the bands observed below 1000 cm^–1^, since these are associated with vibrations involving the metal center. The vibrational modes around 660, 720, 736, and 802 cm^–1^ are attributed to deformations of functional groups, such as carboxylic group and amine group. Even lower, bands exclusively observed in the Raman spectrum nearly 241, 253, 276, 299, and 319 cm^–1^, correspond to metal–ligand vibrational modes, such as Cu–O and Cu–N stretching, and torsions of ligands containing the metal center.^3^ These observations confirm the nature of Cu­(II) coordination and the distorted environment surrounding the metal center.

Vibration bands below 200 cm^–1^ in the Raman spectrum are mainly attributed to the crystalline lattice modes, which can couple to the twisting and deformation movements related to the complex within a crystal structure. These modes are typical of molecular solids and provide information about the overall molecular organization of the crystal. In the [Cu­(l-Ile)­(phen)­(H_2_O)]­Cl·2H_2_O complex, these vibrations reflect not only the packing of the structural units but also involve the presence of an extensive lattice of hydrogen bonds mainly formed by the coordination and crystallization of water molecules. Hydrogen bonds act as flexible bridges between molecules, dynamically coupling the movements of different parts of the crystal structure. This vibrational coupling promotes greater coherence in the lattice vibrational modes and directly influences the position and intensity of the observed bands. Thus, the vibrational modes below 200 cm^–1^ provide important evidence about the intermolecular interactions and the 3D stability of the crystal structure of the complex.^2,3,33^

Taken together, the comparative vibrational analysis of IR and Raman spectra as presented herein proves to be a complementary characterization. While IR is more sensitive to changes in the dipole moment, intensely capturing the vibrations of highly polar groups (such as O–H and C=O), the Raman spectrum excels in detecting vibrations in spectral regions of low wavenumber and involving bonds with low polarity, such as metal–ligand interactions. Thus, the combined vibrational analyses not only allow the identification of functional groups present but also confirm the coordinated structure of the [Cu­(l-Ile)­(phen)­(H_2_O)]­Cl·2H_2_O complex.

### 3.5 Molecular Docking

Molecular docking is a computational analysis that evaluates the pharmacological activity of a synthesized molecule by identifying its minimum potential docking pose and its distinct strong and weak interactions with biomacromolecules.^5,35^ All docking results discussed herein refer exclusively to the cationic complex [Cu­(l-Ile)­(phen)­(H_2_O)]^+^, which represents the species expected to interact with biomacromolecules under physiological conditions. The obtained binding energies and interaction profiles, therefore, reflect a physically realistic model of complex biomolecule interactions.

Figure 7a illustrates the molecular interactions between the [Cu­(l-Ile)­(phen)­(H_2_O)]^+^ complex and DNA, highlighting the coupling mode of the metal with the nucleotide structure. Figure 7b presents a 2D graph detailing the specific interactions between the complex and the nitrogenous bases of DNA, highlighting the points of contact and the types of bonds involved.

**Figure 7:**
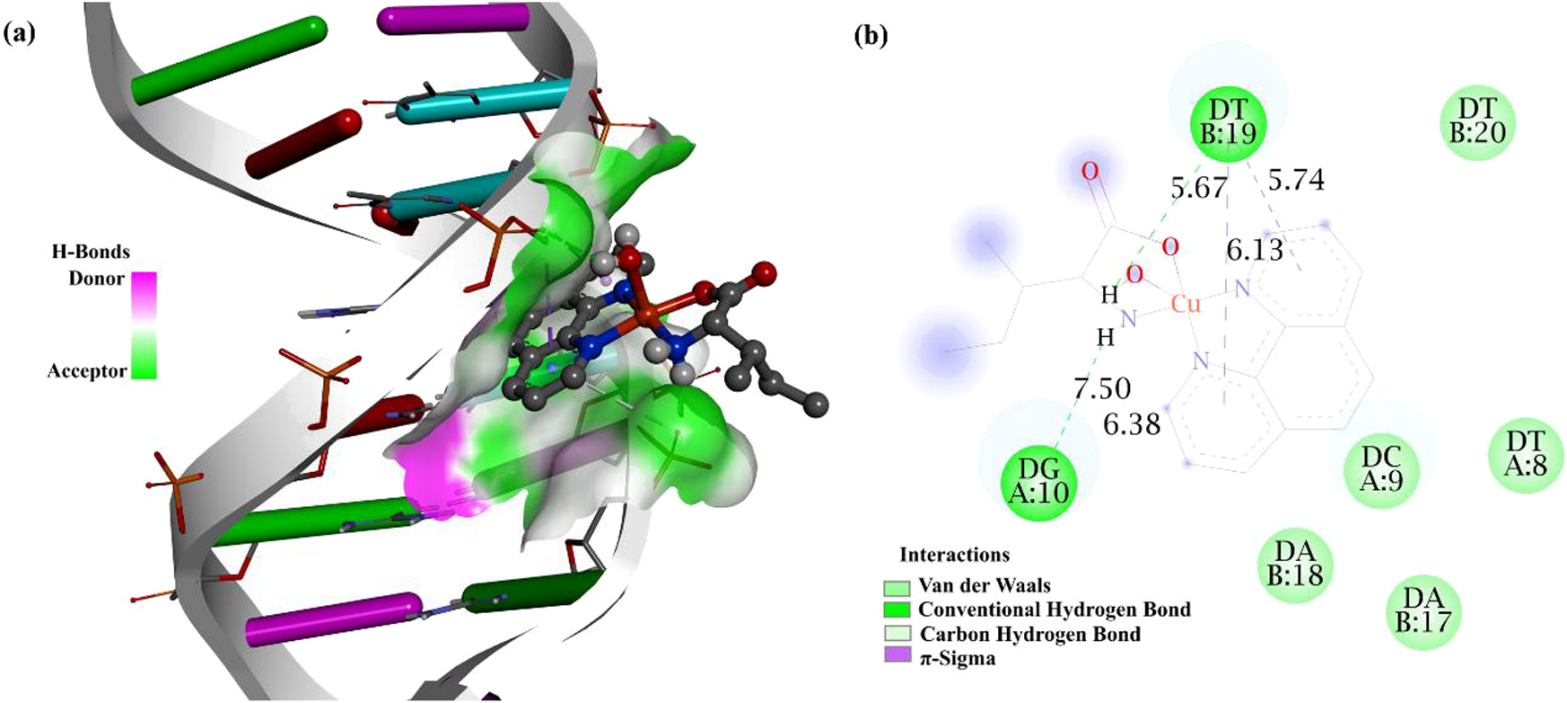
Representation of the [Cu­(l-Ile)­(phen)­(H_2_O)]^+^ complex interacting with DNA: (a) Three-dimensional visualization, and (b) 2D mapping of nucleotide interactions.

The complex fits between the DNA nucleobases, interacting via intercalation, specifically between the phen rings. Thus, the [Cu­(l-Ile)­(phen)­(H_2_O)]^+^ complex fits between the nucleotides without breaking the DNA double helix.^36,37^ The results of the DNA dodecamer yielded a binding energy value of −8.26 kcal/mol. The interactions observed in the 2D graph include van der Waals, conventional hydrogen bonds, carbon–hydrogen bonds, and π-σ interactions. The van der Waals interactions occur through the nucleobases adenine (DA B:18), cytosine (DC A:9), thymine (DT A:8), thymine (DT B:20) and adenine (DA B:17). Conventional hydrogen interactions occur through the thymine (DT B:19), guanine (DG A:10) bases, and the hydrogen atoms belong to the l-Ile molecule and coordinated water. The π-sigma interactions occur between the phen rings and the thymine base (DT B:19).

BSA is the most abundant protein produced in the liver, facilitating the delivery of drugs to their target sites.^38,39^ Figure 8a presents the results of the molecular docking performed with the BSA protein, revealing a promising binding free energy of −7.17 kcal/mol. Figure 8b highlights the most relevant interactions identified based on the binding energy values, including van der Waals interactions, conventional hydrogen bonds, carbon hydrogen bonds, π-alkyl interactions, π–π interactions in T-shaped conformation, and alkyl-type interactions between the protein residues ARG-144, ARG-143, TYR-139, LYS-136, GLU-140, GLU-125, LEU-115, LEU-122, TYR-160 PHE-133 and TYR-137 and the atoms of the phen and l-Ile ligands belonging to the complex.

**Figure 8:**
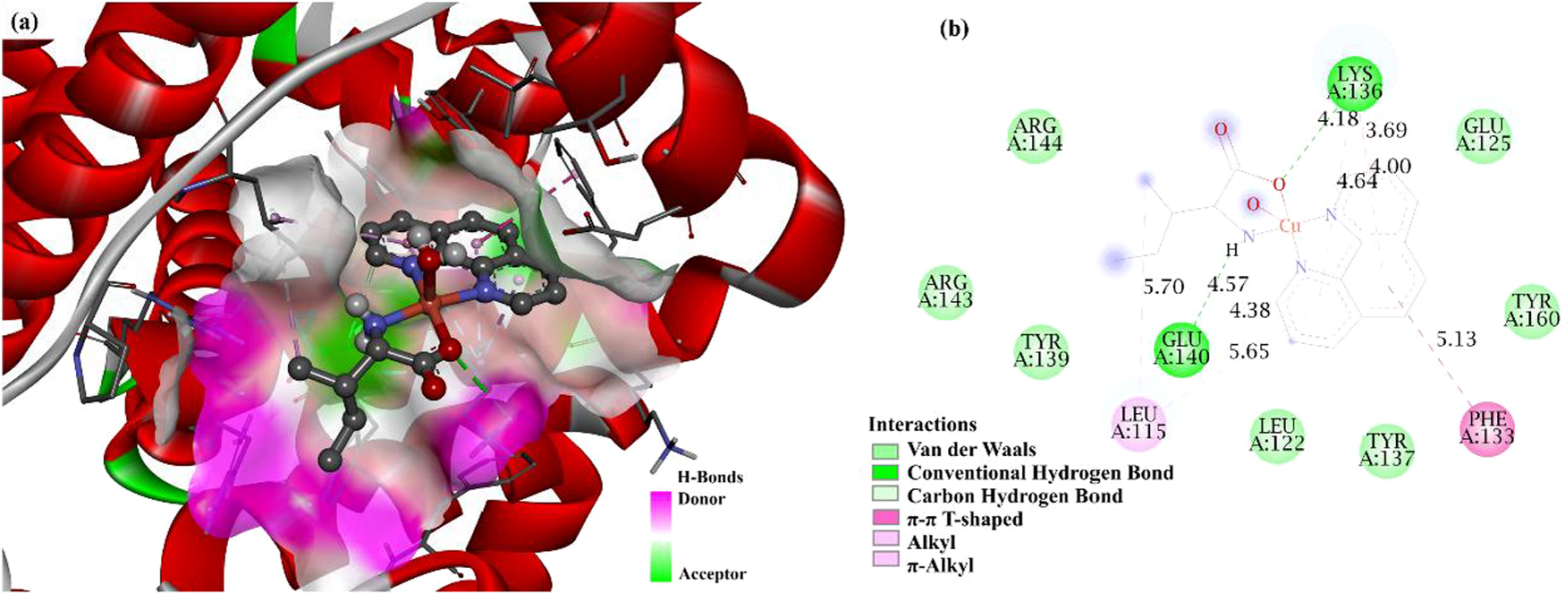
Representation of molecular interaction between the [Cu­(l-Ile)­(phen)­(H_2_O)]^+^ complex and the BSA protein: (a) Three-dimensional docking model and (b) 2D graph of the interactions with the residues.

The resulting molecular interaction of the complex with the DNA dodecamer yielded inhibition constant and binding energy values of 876.42 μM and −8.26 kcal/mol, respectively. For BSA, these were 5.54 μM and −7.17 kcal/mol, respectively. These values show that there is an affinity between the [Cu­(l-Ile)­(phen)­(H_2_O)]^+^ complex and the macromolecules investigated.

Although molecular docking does not provide direct evidence of a biological mechanism, the predicted binding modes and favorable interaction energies support the experimental cytotoxicity data and suggest that DNA and serum albumin may act as relevant molecular targets for this class of Cu­(II)-based complexes.

### 3.6 Cytotoxicity Activity

To investigate the antitumor potential of the newly synthesized Cu­(II) complex, cell viability assays were performed using the cervical tumor cell line SiHa and normal human lung fibroblasts (GM07492A). The [Cu­(l-Ile)­(phen)­(H_2_O)]­Cl·2H_2_O complex produced a dose-dependent cytotoxic effect on SiHa cells (Figure 9a). At the lowest concentrations (0.195 and 0.78 μM), cell viability remained high and comparable to the control, indicating minimal cytotoxicity. From 3.125 μM onward, however, a marked decrease in viability was observed, which became pronounced at 12.5 and 50 μM, demonstrating potent growth inhibition at higher doses. Consistent with these findings, the complex exhibited an IC_50_ of 2.57 μM against SiHa cells, supporting its antiproliferative efficacy against this tumor lineage.

**Figure 9:**
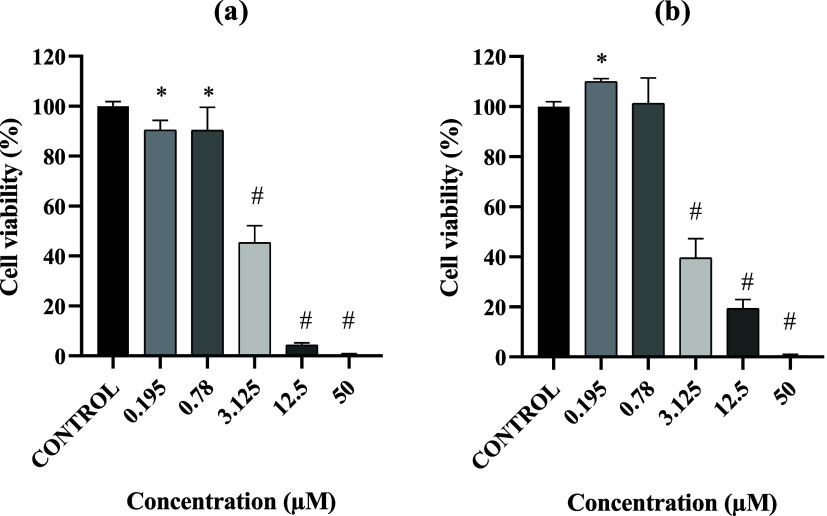
(a) Percentage of cell viability of SiHa cells and (b) percentage of cell viability of GM07492A cells treated with [Cu­(l-Ile)­(phen)­(H_2_O)]­Cl·2H_2_O complex at different concentrations. Statistical analysis was performed using one-way ANOVA followed by Tukey’s post hoc test. Data are expressed as mean ± standard deviation (SD) from two independent experiments. Significant differences compared to the control group (medium only) are indicated by *p < 0.05 and ^#^ *p* < 0.0001.

In GM07492A fibroblasts, the complex also induced cytotoxicity, though to a lesser extent (Figure 9b). At 0.195 and 0.78 μM, cell viability remained above 90% and did not differ significantly from the control (*p > 0.05), indicating that these concentrations are not overtly toxic to normal cells. A pronounced reduction in viability was first observed at 3.125 μM, with values around 60% (^#^ *p* < 0.05); this reduction became even more evident at 12.5 and 50 μM, where viability fell below 20%. The IC_50_ for GM07492A cells was 2.77 μM, yielding a selectivity index of approximately 1.08. Although modest, this SI indicates a detectable tendency for preferential cytotoxicity toward tumor cells. In cytotoxicity studies, SI values ≥2–3 are typically considered indicative of good selectivity, whereas values close to 1 reflect limited differentiation between malignant and nonmalignant cells.^40^ Thus, while the complex displays potent cytotoxicity at low micromolar concentrations, further optimization may enhance its selectivity profile.

Selectivity indices close to 1 indicate limited discrimination between malignant and nonmalignant cells, suggesting that the compound exhibits comparable cytotoxicity in both systems.^41^ Therefore, these findings should be interpreted primarily as a potency-oriented screening outcome rather than definitive evidence of therapeutic selectivity. Although the compound demonstrates biological activity, further structural and pharmaceutical optimization is required to enhance tumor selectivity. Rational strategies may include ligand tuning^42^ as well as formulation-based approaches such as nanoencapsulation or other targeted delivery systems.^43^

As a positive control, cisplatin was evaluated under the same experimental conditions as in the viability assay in the SiHa and GM07492A cell lines, enabling a direct comparison of cytotoxic potency (Figure S1). Both the [Cu­(l-Ile)­(phen)­(H_2_O)]­Cl·2H_2_O complex and cisplatin showed a dose-dependent reduction in cell viability. For cisplatin, IC_50_ values of 15.36 μM were obtained in SiHa and 21.57 μM in GM07492A, resulting in an SI of 1.40. Compared with cisplatin, the complex exhibited greater antiproliferative potency in SiHa (IC_50_ = 2.57 μM) under the same conditions, although with lower selectivity (IS = 1.08), reinforcing its activity at low micromolar concentrations but indicating a need to improve the selectivity profile for nontumor cells.

Copper­(II) complexes containing amino acids, such as tyrosine,^2^ glutamine,^3^ methionine,^4,34^ serine,^5^ and glycine,^6^ have been extensively investigated for their antitumor potential, showing efficacy against several cancer cell lines. These compounds generally act through mechanisms such as the generation of ROS, induction of oxidative stress, and direct interaction with DNA, leading to programmed cell death.^44,45^ In this context, the behavior of [Cu­(l-Ile)­(phen)­(H_2_O)]­Cl·2H_2_O aligns with the cytotoxic effects reported for other amino-acid-based Cu­(II) complexes, particularly regarding its ability to impair tumor cell viability at low micromolar concentrations.

Additionally, unlike complexes coordinated with more polar amino acids, whose transport often depends on active uptake systems, the presence of l-Ile, a nonpolar and hydrophobic amino acid, likely increases the lipophilicity of the complex.^46,47^ This characteristic may facilitate the passive diffusion of [Cu­(l-Ile)­(phen)­(H_2_O)]­Cl·2H_2_O across cell membranes, potentially contributing to the observed cytotoxicity. Such structural considerations, especially those related to ligand hydrophobicity and electronic properties, have been identified as key determinants of the biological activity of copper complexes, influencing both cellular uptake and interactions with intracellular targets.^48^

Furthermore, these findings reinforce the antitumor potential of the [Cu­(l-Ile)­(phen)­(H_2_O)]­Cl·2H_2_O complex, particularly due to its ability to reduce tumor cell viability at low concentrations. While the SI indicates that improvements are still needed to provide greater safety toward normal cells, the dose-dependent pattern and low micromolar IC_50_ values are consistent with the pharmacological behavior expected of promising copper-based antitumor agents.

From a structure–activity relationship perspective, the biological behavior of the [Cu­(l-Ile)­(phen)­(H_2_O)]­Cl·2H_2_O complex can be rationalized by the complementary roles of its ligands. The planar and aromatic phen fragment favors π–π stacking and intercalative interactions with DNA, as supported by molecular docking results. In contrast, the presence of l-Ile, a branched and hydrophobic amino acid, is expected to increase the complex’s overall lipophilicity, facilitating passive diffusion across cell membranes. The Cu­(II) center also contributes through redox activity, which is commonly associated with the generation of reactive oxygen species (ROS) in copper-based antitumor systems.^3,7^ Together, these structural features provide a coherent explanation for the observed low-micromolar cytotoxicity and establish a clear structure–activity relationship for this mixed-ligand Cu­(II) complex.

## 4 Conclusions

A ternary Cu­(II) complex, [Cu­(l-Ile)­(phen)­(H_2_O)]­Cl·2H_2_O, was successfully synthesized and structurally characterized by SCXRD, revealing a distorted square-pyramidal coordination geometry in a monoclinic (*P*2_1_). Hirshfeld surface analysis demonstrated that the crystal packing is dominated by H···H (52.8%), O···H/H···O (17.4%), and Cl···H/H···Cl (10.3%) interactions, highlighting the role of the chloride counterion and water molecules in supramolecular stabilization. Molecular docking studies conducted with the cationic species [Cu­(l-Ile)­(phen)­(H_2_O)]^+^ predicted favorable binding to DNA (Δ*G* = −8.26 kcal/mol) and bovine serum albumin (Δ*G* = −7.17 kcal/mol), supported by π–π and electrostatic interactions. *In* *vitro* assays revealed low-micromolar antiproliferative activity against SiHa cervical carcinoma cells (IC_50_ = 2.57 μM) with limited selectivity relative to nontumor fibroblasts (SI = 1.08). Taken together, this study establishes a validated Cu­(II) mixed-ligand platform and highlights the importance of integrating structural, supramolecular, computational, and biological data when assessing the biomedical potential of coordination compounds.

## Supplementary Material



## Notes

The cytotoxic effect of cisplatin (Figure S1), crystallographic data, refinement details, fractional atomic coordinates and anisotropic displacement parameters (Tables S1 and S2), hydrogen-bond geometry (Table S3), torsion angles (Table S4), and additional structural parameters of hydrogen atoms (Table S5). (PDF)
