# Supplementary material for: Unlocking Biomedical Potential of the [Cu(l‑isoleucine)(1,10-phenanthroline)(H2O)]Cl·2H2O Complex: A Comprehensive Study of Synthesis, Spectroscopic Analyses, Antitumor Efficacy, and Computational Insights
Source: ACS Omega. 2026 May 14;11(20):29772–86. doi: 10.1021/acsomega.6c00071 (PMC13216970; doi:10.1021/acsomega.6c00071)
Supplement: Supplementary file 1 [file ao6c00071_si_001.pdf]

Supporting Information

Unlocking biomedical potential of the [Cu(L-isoleucine)(1,10-phenanthroline)(H_2O)]Cl□2H_2O complex: a comprehensive study of synthesis, spectroscopic analyses, antitumor efficacy, and computational insights

Marinaldo V. de Souza Junior,[§,#] Jayson C. dos Santos,[§] Jad L. F. Simplicio,[§,#] João G. de Oliveira Neto,[§] Camila P. S. Silva,[⊥] Aramys S. Reis,[⊥] Adenilson O. dos Santos,[§] Alejandro P. Ayala,[‡] Eliana B. Souto,[#,*] Francisco F. de Sousa[§,†,*]

[§]Center for Sciences of Imperatriz, Federal University of Maranhão – UFMA, 65900-410, Imperatriz, MA, Brazil.
[#]UCD School of Chemical and Bioprocess Engineering, University College Dublin, Belfield, Dublin 4, D04 V1W8, Ireland.
[⊥]Laboratory of Pathophysiology and Therapeutic Research, Center for Social Sciences, Health and Technology, Federal University of Maranhão - UFMA, 65900-410, Imperatriz, MA, Brazil.
[‡]Department of Physics, Federal University of Ceará – UFC, Fortaleza, Ceará, CEP 65455-900, Brazil.
[†]Institute of Exact and Natural Sciences, Federal University of Pará – UFPA, 66075-110, Belém, PA, Brazil.

Corresponding authors.

[*]Eliana B. Souto, UCD School of Chemical and Bioprocess Engineering, University College Dublin, Belfield, Dublin 4, D04 V1W8, Ireland. **E-mail**: eliana.souto@ucd.ie

[*]Francisco F. de Sousa, Institute of Exact and Natural Sciences, Federal University of Para – UFPA, 66075-110, Belém, PA, Brazil. **E-mail**: ffs@ufpa.br

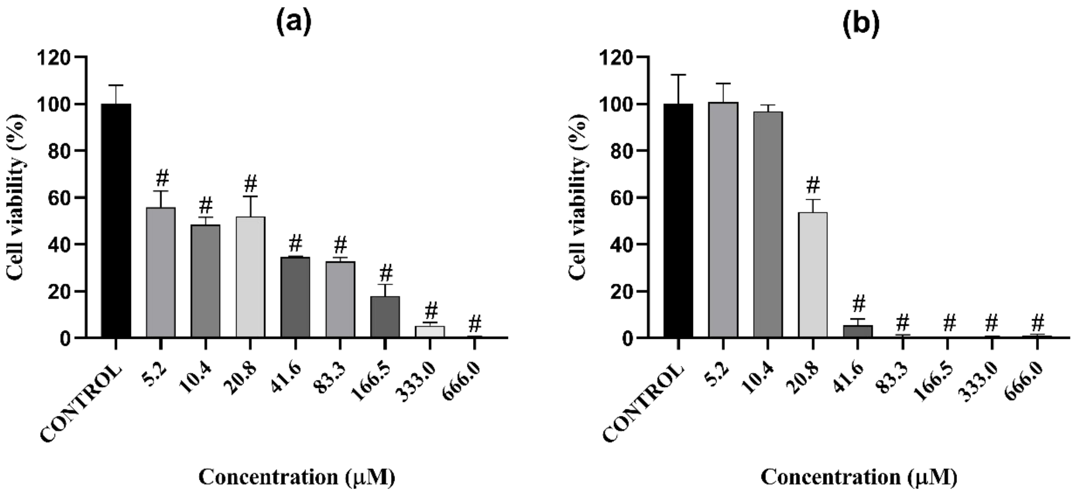

Figure S1. Cytotoxic effect of cisplatin (positive control) on (a) SiHa (cervical carcinoma cells) and (b) GM07492A (human fibroblasts) cell lines after 72 h of incubation, determined by the MTT assay.

Table S1. Fractional Atomic Coordinates ($\times 10^4$) and Equivalent Isotropic Displacement Parameters ($Å^2 \times 10^3$) for $[Cu(L\text{-}Ile)(phen)(H_2O)]Cl\square 2H_2O$. U_{eq} is defined as 1/3 of the trace of the orthogonalised U_{IJ} tensor.

Atom	*x*	*y*	*z*	U(eq)
**Cu1**	7114.6(3)	5185.5(8)	3285.0(2)	44.2(13)
**Cl2**	5453.7(11)	-334.5(14)	1662.1(9)	74.2(3)
**O1**	8783.0(2)	5903.0(4)	3745.6(18)	53.1(6)
**O4**	7522.0(3)	2095.0(4)	3095(2)	64.4(7)
**N2**	7002.0(2)	5097.0(6)	4694.6(19)	47.1(5)
**N1**	5330.0(2)	5072.0(6)	2921.0(2)	50.9(6)
**O2**	10373(2)	5770.0(7)	3258.0(3)	92.0(15)
**N3**	7243.0(2)	5824.0(5)	1932.0(2)	49.3(7)
**O5**	9038.0(3)	-203.0(5)	4506.0(3)	85.3(11)
**C6**	5855.0(3)	5062.0(7)	4708.0(2)	45.9(6)
**C13**	9293.0(3)	5774.0(6)	3063.0(3)	55.1(9)
**C5**	4962.0(3)	5065.0(7)	3753.0(3)	47.6(6)
**C14**	8492.0(3)	5566.0(6)	1970.0(3)	53.2(10)
**C7**	5554.0(3)	5039.0(7)	5602.0(3)	56.3(8)
**C4**	3754.0(3)	5089.0(8)	3696.0(3)	59.7(8)
**C12**	7862.0(3)	5071.0(9)	5575.0(3)	58.5(8)
**C1**	4516.0(3)	5094.0(9)	2011.0(3)	63.7(8)
**C2**	3290.0(3)	5157.0(11)	1896.0(4)	77.2(11)

**O3**	6203.0(5)	2942.0(8)	403.0(3)	132.0(2)
**C15**	8870.0(4)	6883.0(7)	1229.0(3)	66.8(11)
**C11**	7629.0(4)	5035.0(11)	6494.0(3)	70.6(10)
**C3**	2924.0(3)	5135.0(9)	2718.0(4)	73.1(11)
**C17**	8879.0(5)	9006.0(8)	1516.0(5)	88.1(17)
**C8**	4312.0(4)	5046.0(8)	5514.0(4)	69.8(11)
**C10**	6479.0(4)	5022.0(9)	6509.0(3)	68.1(10)
**C9**	3463.0(4)	5081.0(9)	4611.0(4)	70.5(11)
**C16**	8123.0(5)	6446.0(11)	143.0(4)	93.1(18)
**C18**	9523.0(5)	10306.0(14)	967.0(5)	111.0(2)

Table S2. Anisotropic displacement parameters ($Å^2 \times 10^3$) for [Cu(L-Ile)(phen)(H_2O)]Cl□2H_2O. The anisotropic displacement factor exponent takes the form: $-2\pi^2[h^2a^{\times 2}U_{11}+2hka^{\times}b^{\times}U_{12}+\dots]$.

Atom	U_{11}	U_{22}	U_{33}	U_{23}	U_{13}	U_{12}
**Cu1**	36.1(19)	53.2(2)	44.9(19)	0.6(2)	14.8(13)	-1.6(19)
**Cl2**	74.2(7)	66.6(8)	71.2(6)	-6.3(4)	7.3(5)	-5.3(4)
**O1**	43.3(13)	67.1(15)	48.6(12)	-1.5(11)	13.8(10)	-7.8(10)
**O4**	65.9(17)	54.6(15)	63.9(16)	-3.3(12)	7.4(13)	0.9(12)
**N2**	44.3(12)	48.7(13)	51.4(12)	1.3(17)	19.3(10)	1.0(16)
**N1**	38.8(12)	54.0(15)	59.9(14)	0.4(19)	15.1(10)	-1.5(17)
**O2**	37.5(14)	158.0(5)	80.8(19)	11.0(2)	18.0(13)	10.1(18)
**N3**	38.7(14)	62.7(18)	47.3(14)	5.6(12)	14.3(11)	1.0(11)
**O5**	86.0(2)	71.0(3)	75.4(19)	-7.5(17)	-9.8(16)	6.3(17)
**C6**	50.5(15)	35.5(14)	60.1(16)	0.3(19)	29.1(13)	4.7(17)
**C13**	38.5(17)	68.0(3)	60.0(2)	6.9(16)	17.4(15)	3.8(14)
**C5**	41.1(14)	34.6(14)	71.3(18)	-2.0(2)	23.7(13)	-0.4(17)
**C14**	45.1(17)	67.0(3)	51.4(17)	6.1(16)	20.6(14)	10.3(15)
**C7**	73.0(2)	37.3(16)	73.0(2)	2.0(2)	43.1(18)	2.0(2)
**C4**	46.2(16)	40.4(15)	101.0(3)	-1.0(3)	35.6(17)	1.0(2)
**C12**	51.1(17)	69.0(2)	54.8(17)	2.0(2)	15.9(14)	1.0(2)
**C1**	45.0(16)	73.0(2)	65.5(19)	0.0(3)	6.2(14)	-3.0(3)
**C2**	45.3(18)	81.0(3)	92.0(3)	-11.0(4)	3.4(18)	3.0(3)
**O3**	172.0(5)	123.0(4)	65.0(2)	15.0(2)	-14.0(3)	-51.0(4)
**C15**	55.0(2)	90.0(3)	68.0(2)	19.0(2)	38.0(2)	15.0(2)
**C11**	82.0(3)	75.0(3)	52.7(18)	4.0(3)	17.9(17)	3.0(3)
**C3**	36.2(16)	62.0(2)	117.0(3)	-7.0(3)	17.3(18)	-1.0(2)
**C17**	84.0(4)	87.0(4)	114.0(4)	24.0(3)	61.0(3)	1.0(3)
**C8**	82.0(3)	51.0(2)	103.0(3)	5.0(3)	68.0(3)	5.0(2)
**C10**	96.0(3)	58.0(2)	65.0(2)	4.0(2)	46.0(2)	6.0(3)
**C9**	60.0(2)	55.0(2)	114.0(3)	1.0(3)	52.0(2)	0.0(3)

**C16**	90.0(4)	139.0(5)	60.0(3)	24.0(3)	39.0(3)	17.0(4)
**C18**	102.0(4)	113.0(5)	134.0(5)	45.0(6)	59.0(4)	-2.0(5)

Table S3. Hydrogen bonds for [Cu(L-Ile)(phen)(H_2O)]Cl□2H_2O.

D	H	A	d(D-H) (Å)	d(H-A) (Å)	d(D-A) (Å)	D-H-A (°)
**O4**	**H4A**	**O5**	0.86	1.95	2.72(4)	147.60
**N3**	**H3A**	**O3**	0.89	2.03	2.89(6)	162.30
**O5**	**H5B**	**O1**[1]	0.85	2.10	2.87(5)	151.50

[1]+X,-1+Y,+Z

Table S4. Torsion angles for [Cu(L-Ile)(phen)(H_2O)]Cl□2H_2O.

Atom				Angle (°)	Atom				Angle (°)
**Cu1**	**O1**	**C13**	**O2**	-164.3(4)	**C6**	**C5**	**C4**	**C9**	-0.9(8)
**Cu1**	**O1**	**C13**	**C14**	14.1(4)	**C6**	**C7**	**C8**	**C9**	-0.2(8)
**Cu1**	**N2**	**C6**	**C5**	0.9(5)	**C6**	**C7**	**C10**	**C11**	0.2(8)
**Cu1**	**N2**	**C6**	**C7**	-178.6(4)	**C13**	**C14**	**C15**	**C17**	-58.3(5)
**Cu1**	**N2**	**C12**	**C11**	179.1(5)	**C13**	**C14**	**C15**	**C16**	173.8(4)
**Cu1**	**N1**	**C5**	**C6**	-2.3(5)	**C5**	**N1**	**C1**	**C2**	1.5(8)
**Cu1**	**N1**	**C5**	**C4**	176.7(4)	**C5**	**C6**	**C7**	**C8**	-0.8(7)
**Cu1**	**N1**	**C1**	**C2**	-174.9(5)	**C5**	**C6**	**C7**	**C10**	179.5(5)
**Cu1**	**N3**	**C14**	**C13**	-24.2(4)	**C5**	**C4**	**C3**	**C2**	-0.1(9)
**Cu1**	**N3**	**C14**	**C15**	-151.4(3)	**C5**	**C4**	**C9**	**C8**	-0.1(8)
**O1**	**C13**	**C14**	**N3**	7.5(5)	**C14**	**C15**	**C17**	**C18**	166.2(5)
**O1**	**C13**	**C14**	**C15**	135.2(4)	**C7**	**C6**	**C5**	**N1**	-179.5(4)
**N2**	**C6**	**C5**	**N1**	1.0(7)	**C7**	**C6**	**C5**	**C4**	1.4(7)
**N2**	**C6**	**C5**	**C4**	-178.1(5)	**C7**	**C8**	**C9**	**C4**	0.7(9)
**N2**	**C6**	**C7**	**C8**	178.6(5)	**C12**	**N2**	**C6**	**C5**	-179.2(5)
**N2**	**C6**	**C7**	**C10**	-1.0(7)	**C12**	**N2**	**C6**	**C7**	1.3(7)
**N2**	**C12**	**C11**	**C10**	0.1(10)	**C12**	**C11**	**C10**	**C7**	0.2(10)

**N1**	**C5**	**C4**	**C3**	-0.4(7)	**C1**	**N1**	**C5**	**C6**	-179.4(5)
**N1**	**C5**	**C4**	**C9**	-180.0(5)	**C1**	**N1**	**C5**	**C4**	-0.3(7)
**N1**	**C1**	**C2**	**C3**	-2.1(10)	**C1**	**C2**	**C3**	**C4**	1.3(10)
**O2**	**C13**	**C14**	**N3**	-174.0(4)	**C3**	**C4**	**C9**	**C8**	-179.6(6)
**O2**	**C13**	**C14**	**C15**	-46.4(5)	**C8**	**C7**	**C10**	**C11**	-179.4(6)
**N3**	**C14**	**C15**	**C17**	67.0(5)	**C10**	**C7**	**C8**	**C9**	179.4(6)
**N3**	**C14**	**C15**	**C16**	-60.9(5)	**C9**	**C4**	**C3**	**C2**	179.4(6)
**C6**	**N2**	**C12**	**C11**	-0.8(9)	**C16**	**C15**	**C17**	**C18**	-68.0(6)
**C6**	**C5**	**C4**	**C3**	178.6(5)					

Table S5. Hydrogen atom coordinates (Å×10^4) and isotropic displacement parameters (Å^2×10^3) for [Cu(L-Ile)(phen)(H_2O)]Cl□2H_2O.

**Atom**	*x*	*y*	*z*	**U(eq)**
**H4A**	7838.84	1562.51	3679.28	97.00
**H4B**	6873.63	1451.10	2838.39	97.00
**H3A**	6775.66	5046.29	1464.66	59.00
**H3B**	7016.41	7041.21	1770.20	59.00
**H5A**	9372.18	104.75	5121.95	128.00
**H5B**	9133.63	-1420.71	4489.13	128.00
**H14**	8571.80	4227.96	1766.36	64.00
**H12**	8646.90	5078.06	5576.04	70.00
**H1**	4760.32	5067.70	1436.39	76.00
**H2**	2738.53	5213.76	1251.74	93.00
**H3C**	5574.53	3619.71	198.34	197.00
**H3D**	6049.73	1885.17	651.00	197.00
**H15**	9690.13	6536.33	1286.87	80.00
**H11**	8252.38	5018.89	7098.89	85.00
**H3**	2116.16	5150.41	2641.54	88.00
**H17A**	9258.29	9132.63	2241.03	106.00

**H17B**	8066.16	9449.75	1365.36	106.00
**H8**	4091.64	5026.14	6100.25	84.00
**H10**	6318.06	5001.94	7123.56	82.00
**H9**	2669.21	5098.98	4585.63	85.00
**H16A**	8138.61	5079.60	20.60	140.00
**H16B**	8440.37	7130.64	-316.22	140.00
**H16C**	7319.38	6851.26	40.55	140.00
**H18A**	9163.79	10170.87	248.10	167.00
**H18B**	10343.20	9934.58	1149.00	167.00
**H18C**	9466.78	11629.63	1157.73	167.00
